# Recent Advances on Detection and Characterization of Fruit Tree Viruses Using High-Throughput Sequencing Technologies

**DOI:** 10.3390/v10080436

**Published:** 2018-08-17

**Authors:** Varvara I. Maliogka, Angelantonio Minafra, Pasquale Saldarelli, Ana B. Ruiz-García, Miroslav Glasa, Nikolaos Katis, Antonio Olmos

**Affiliations:** 1 Laboratory of Plant Pathology, School of Agriculture, Faculty of Agriculture, Forestry and Natural Environment, Aristotle University of Thessaloniki, 54124 Thessaloniki, Greece; katis@agro.auth.gr; 2 Istituto per la Protezione Sostenibile delle Piante, Consiglio Nazionale delle Ricerche, Via G. Amendola 122/D, 70126 Bari, Italy; angelantonio.minafra@ipsp.cnr.it (A.M.); pasquale.saldarelli@ipsp.cnr.it (P.S.); 3 Centro de Protección Vegetal y Biotecnología, Instituto Valenciano de Investigaciones Agrarias (IVIA), Ctra. Moncada-Náquera km 4.5, 46113 Moncada, Valencia, Spain; ana.belen.ruiz@uv.es (A.B.R.-G.); aolmos@ivia.es (A.O.); 4 Institute of Virology, Biomedical Research Centre, Slovak Academy of Sciences, Dúbravská cesta 9, 84505 Bratislava, Slovak Republic; Miroslav.Glasa@savba.sk

**Keywords:** high-throughput sequencing, fruit trees, new viruses, detection, variability, siRNAs, total RNA, dsRNA

## Abstract

Perennial crops, such as fruit trees, are infected by many viruses, which are transmitted through vegetative propagation and grafting of infected plant material. Some of these pathogens cause severe crop losses and often reduce the productive life of the orchards. Detection and characterization of these agents in fruit trees is challenging, however, during the last years, the wide application of high-throughput sequencing (HTS) technologies has significantly facilitated this task. In this review, we present recent advances in the discovery, detection, and characterization of fruit tree viruses and virus-like agents accomplished by HTS approaches. A high number of new viruses have been described in the last 5 years, some of them exhibiting novel genomic features that have led to the proposal of the creation of new genera, and the revision of the current virus taxonomy status. Interestingly, several of the newly identified viruses belong to virus genera previously unknown to infect fruit tree species (e.g., *Fabavirus*, *Luteovirus*) a fact that challenges our perspective of plant viruses in general. Finally, applied methodologies, including the use of different molecules as templates, as well as advantages and disadvantages and future directions of HTS in fruit tree virology are discussed.

## 1. Introduction

Fruit trees host a wide range of viral pathogens, mainly as a result of their vegetative mode of propagation and perennial nature [1]. Notably, among fruit trees, 44 viruses have been identified in the major cultivated *Prunus* species [2]. Some of these pathogens are causing diseases adversely affecting yield and/or decreasing the fruit quality [1]. The identification, detection, and characterization of the causal agents of such diseases are challenging, due to the low titer and irregular within-plant distribution of viruses in perennial plants, the occurrence of both inter- and intra-species mixed-infections in single trees, the frequent symptomless infections or fluctuation of symptom intensity during the season, and the complex and heterogeneous nature of viral populations [3,4,5,6,7,8,9].

Several biological, serological, and molecular assays have been applied, either alone or in combination, for the detection of fruit tree viruses. Biological assays, also known as biological indexing, were, in the past, an essential tool for the evaluation of the health status of individual plants and for the detection and/or characterization of a particular virus. In the case of fruit tree viruses, bioassays took advantage of the increased susceptibility of some fruit tree genotypes to viral infection, resulting in pronounced and easily identifiable symptoms expression. A number of fruit tree genotypes have been used for this purpose, e.g., *Malus platycarpa*, *Malus micromalus*, *Malus pumila* “Virginia Crab” or “Spy227”, for apple and pear virus pathogens [10,11]; and *Prunus persica* GF305, *Prunus tomentosa*, *Prunus serrulata* “Kwanzan” or “Shirofugen” [12,13,14], for stone fruit viral pathogens. Besides woody indicators, several herbaceous plant species, showing either local or systemic symptoms, have also been used [3,10,15], although no universal indicators for all fruit viruses are available.

Effectiveness of bioassays vary considerably and can be hampered by several factors. Firstly, there is a need for controlled greenhouse facilities for plant maintenance, due to the vector-mediated transmission of several viruses, thus limiting a large scale and easy-to-use evaluation. Although symptoms on woody indicators could appear a few weeks post-inoculation, they may take from several months to years in the case of some viruses (e.g., *Plum pox virus*, PPV). In addition, a high intra-species genetic diversity of fruit tree viruses may result, not only in variable symptomatology [16,17], but also in symptomless reactions (e.g., PPV-Rec isolates in GF305 peach seedlings) [18]. However, a question arises concerning the health status of used indicators, if not issued from in vitro meristem culture or thermotherapy sanitation, due to the possibility of hidden infections possibly influencing the interpretation of results.

Nowadays, although still necessary in fruit tree virus research (assessment of biological properties, pathotyping etc.) and certification schemes, the labor- and time-consuming biological indexing, with problematic reliability and subjective evaluation, has less practical application in virus diagnosis.

Serological methods, ELISA (enzyme-linked immunosorbent assay) and its modifications, although limited in sensitivity, enable cheap and fast parallel diagnosis of many samples, using either polyclonal, monoclonal, or recombinant antibodies [19,20,21]. However, immunogenicity of some fruit tree viruses is scarce, producing antibodies which are not reliable enough for mass-scale diagnosis. Therefore, specific and sensitive detection of most fruit tree viruses and virus-like pathogens is mainly based on molecular diagnosis.

Generally, detection of viral pathogens by genome amplification techniques (Polymerase chain reaction: PCR, Reverse transcription polymerase chain reaction: RT-PCR and Loop-mediated isothermal amplification: LAMP) is more sensitive than ELISA, while offering the possibility of multiple detection [22,23,24,25]. However, the development of specific molecular tests is strongly dependent on the knowledge of the pathogen genome and its molecular diversity, making primer design highly dependent on (or limited by) available sequence datasets. Especially in the case of recently discovered or poorly studied viruses, the available sequences might not represent the real pathogen diversity present in the field, resulting in the development of insufficiently polyvalent tests, which might provide false negative results. Similar situations also occurred with well-known and widely spread viruses, such as *Prune dwarf virus* (PDV) [26] and *Apple chlorotic leaf spot virus* (ACLSV) [27], highlighting the need for continuous assessment of the viral molecular variability as a prerequisite to develop polyvalent and efficient detection tools.

Such problems can be overcome by the use of modern next generation sequencing (NGS) or high-throughput sequencing (HTS) technologies, which, theoretically, can produce sequence data of every putative viral agent present in a sample without the need of any former knowledge about their genome [28]. This fact, in addition to the relatively little time needed for the completion of a sequence run and the analysis of the resulting data, make HTS the undisputed leading technology in diagnostics, nowadays.

## 2. Principles and Technologies of High-Througput Sequencing

HTS technologies were introduced in 2005 with the commercialization of FLX Genome Sequencer from 454 Life Sciences using real-time sequencing-by-synthesis pyrosequencing technology [29]. The 454 Life Sciences platform was discontinued by Roche in 2016, as it was eclipsed by newer and more competitive platforms. However, this approach established the basis for current PCR-based HTS technologies. Applied Biosystems SOLiD platform was commercialized in 2006, and it was based on a different technology: Sequencing by oligo ligation. These sequencers were also discontinued in 2016. Currently, Illumina and Ion Torrent platforms are the only technologies available in the category of PCR-based HTS.

Nowadays, Illumina offers nine different sequencers (Table 1) that can produce from 4 million to 20 billion short reads, from 50 to 300 nucleotides. It is the most used technology in fruit tree virus research, and couples sequencing-by-synthesis on a surface of a flow cell divided in lanes. Oligonucleotides complementary to the specific adapters ligated to the library DNA fragments are covalently attached to the surface. PCR occurs in the flow cell through a process that includes heating–cooling and incubation with PCR reagents, thus, producing millions of clusters that are generated from single nucleic acid fragments. Sequencing is performed using the four nucleotides labelled with different fluorescent dyes, blocked chemically at their 3′-end, to allow only the incorporation of one nucleotide. After excitation and image capture, the fluorescent dye is chemically unblocked and removed, allowing the incorporation of the next nucleotide in the next cycle. Successively, low quality reads are filtered out [30]. Illumina sequencers can exploit different strategies for HTS analysis of plant viruses, shotgun approach from total DNA or RNA (RNASeq), nucleic acid sequencing from partially or totally purified viral particles, and sequencing of small interfering RNAs (siRNAs).

Ion Torrent platform was introduced in 2010 by Life Technologies. The first commercialized sequencer was the Ion Personal Genome Machine (PGM), which added a revolutionary semiconductor sequencing technology. Ion Torrent™ Technology directly translates chemical output into digital information on a semiconductor chip. The technology exploits the biochemical process that takes place when the polymerase incorporates a nucleotide into the growing DNA strand when a hydrogen ion (H^+^) is released as a byproduct. Thus, the technology is based on the real time detection of hydrogen ion (H^+^) concentration, representing a change in the pH due to the ion release when a nucleotide is incorporated into a DNA strand. The principal components of the Ion Torrent sequencers are the sequencing chips. These microprocessor chips incorporate extremely dense arrays of millions of micromachined wells coupled to a proprietary ion sensor. Each well is able to hold a single DNA molecule from the DNA library. Currently, Ion Torrent commercializes three different sequencers that can hold different types of chips (Table 1). Ion Torrent sequencers can produce from 550,000 to 100 million short reads, from 200 to 400 nucleotides. As in the case of Illumina, Ion Torrent platform can exploit RNASeq, nucleic acid sequencing from partially or totally purified viral particles, and siRNA sequencing.

Other novel technologies based on single molecule, real-time (SMRT) sequencing (Pacific Biosciences) where no amplification step for sample preparation is required, are in development. Pacific Biosciences offers two different sequencers that can generate from 500,000 to 10 million reads, with an average length of 1000 nucleotides (Table 1). Recently, Oxford Nanopore has developed the MinION technology, based on the use of nanopores, a platform smaller than a smartphone that runs by plugging it through an USB connection to the laptop or computer. Currently, there are three sequencers from Oxford Nanopore (Table 1). The SMRT sequencing has been applied for detection and identification of human pathogens, viruses [31], and bacteria [32]. In plant pathology, this technology is still in development, and its application for fruit tree viruses detection remains to be studied.

## 3. High-Throughput Sequencing Methodology Applied to the Detection of Fruit Tree Viruses 

### 3.1. Sample Preparation

Sample preparation for HTS usually includes a set of common steps, regardless of the HTS platform: Nucleic acid extraction, fragmentation, and/or size-selection; enrichment of RNA viral sequences; preparation of libraries; and bioinformatic analysis. Although these procedures require significant manipulation, time, and skills on genomic library preparation, currently, there are commercially available automated systems able to produce sequence-ready nucleic acids libraries. Platform manufacturers and third-party companies provide specific kits simplifying and standardizing the construction of libraries that, in addition, can be prepared for multiplexing if necessary. Optimal results are obtained using highly pure starting nucleic acids preparation. Contamination, inhibitors, or impurities in the starting material can produce false negative results and artifacts. Although nucleic acid purification, even from woody plants, is a routine task in many laboratories, the preparation of high quality nucleic acids for HTS is not trivial. Different types of targets have been used for fruit tree virus detection, as follows. 

(a) Virus-derived small interfering RNAs 

RNA silencing constitutes a major antiviral mechanism in plants, where hosts enzymes called dicers cut the viral RNA into small interfering RNAs (siRNAs) 21–24 nt long. These molecules can be isolated from the infected plants, subjected to HTS, and used to reconstruct viral genomes. Virus-derived siRNAs have been used to detect known and unknown plant viruses in fruit trees by HTS [33,34,35,36,37,38,39,40,41,42]. The advantage of this approach is that siRNAs can be used to detect RNA and DNA viruses, and also, to detect integrated endogenous viral elements (EVEs), as long as they are transcribed. Moreover, it has been successfully used to recover a persistent virus in persimmon [37]. The disadvantage of this approach is the small size of the viral reads that may complicate the genome assembling process, especially for novel viruses. 

(b) Double-stranded RNA 

dsRNA has been exploited to detect fruit tree viruses by HTS, and also in the study of diseases of unknown etiology [40,43,44,45,46,47,48,49,50]. The advantage of using this target for HTS is an enrichment of viral nucleic acids sequences. Current protocols rely on phenol/chloroform extraction to obtain a mix of total DNA and RNA, from which the dsRNA fraction is enriched by chromatography on a long polymer cellulose matrix [51]. Although the detection of DNA viruses can be scarce, dsRNA has facilitated the discovery of many new RNA viruses, such as *Apricot vein clearing associated virus* (AVCaV) [44], *Prunus virus T* (PrVT) [45], a new luteovirus species provisionally named Cherry associated luteovirus (ChALV) [48], or a new fabavirus named Cherry virus F [40].

(c) Total DNA or RNA

Total DNA-sequencing has been scarcely used in the detection of viruses from woody plants. It has been successfully applied in citrus to identify a new DNA virus [52] using genomic DNA as target, which was subsequently submitted to fragmentation to prepare Illumina sequencing-compatible libraries. 

Total RNA-sequencing has been used to detect and characterize viruses in several woody crops, such as grapevine [53,54,55,56], apple, pear [57], and cherry [9]. It has also been successfully applied for the discovery of two new fabaviruses infecting *Prunus* species [40,49]. Although traditional total RNA purification can be performed, there are commercially available kits that can be used to substitute in-house purifications. A potential drawback of the method is that viral titer can be low within the background of plant RNA, a limitation that can be overcome by depletion of highly abundant plant ribosomal RNA from the total RNA pool.

(d) Virion-associated nucleic acids purified from virus-like particles

Virion-associated nucleic acids (VANA) are used to enrich viral nucleic acids by purifying viral particles from the plant material [58], however, to date, there is no report of the application of the method to fruit tree viruses. The disadvantage of this method is the requirement of complex sample processing. In addition, it is limited to the detection of encapsidated viruses.

### 3.2. Bioinformatics

The bioinformatic analysis of the HTS data can be performed with biologist-friendly commercially available packages or with open platforms. Quality control, sequence assembly, contig annotation, and identification of variations between samples are required steps for the analysis of data. Quality control depends on the technology and the standard parameters whose thresholds are usually provided by the manufacturer. The assembly of reads to recover a viral genome can rely on two different approaches: (i) De novo assembly, where the viral genomes are partially or fully reconstructed by annotation of the generated contigs, or (ii) reads mapping against a reference sequence. For low titer fruit tree viruses, a combination of both approaches might be required, along with higher sequencing depth. Annotation of contigs is performed using BLASTn, BLASTx, BLASTp, and tBLASTx (https://blast.ncbi.nlm.nih.gov/Blast.cgi). De novo assembly has limitations, especially when faced with the recovery of several viral variants or virus strains of the same virus species in a single plant sample. This occurs particularly when siRNAs are used as template for HTS, due to the low length of reads that can produce false positive results or incorrect assembly. In that case, a strategy that uses longer reads (e.g., 100 nucleotides or higher) can help solve the problem, as stringent assembly parameters facilitates the recovery of each of the isolates that infect the sample. Subsequently, different nucleotide sequences among variants, such as single nucleotide polymorphism (SNP) or insertions and deletions (indels), can be analyzed by PCR amplification and restriction fragment length polymorphism (RFLP) analysis. The increasing number of available host genomes allows for the bioinformatic depletion of host genomes that, along with the deduplication of reads, can facilitate the analysis at high sequencing depth. There is a continuous development of suitable pipelines for plant virus discovery and detection, in the attempt to reconcile computing needs with the pace of the improvements and development of new HTS platforms. The first automatic pipeline for the identification of viruses in RNA-sequenced samples, VirusFinder, was developed by Wang et al. [59]. A detailed comparison between this and other available automatized bioinformatics tools, VirusHunter, VirFind, ezVIR, ViromeScan, Taxonomer, VIP, VirusDetect, VSD toolkit, and Metavisitor, has been reported by Jones et al. [60].

## 4. Application of High-Throughput Sequencing Technology to Detection and Discovery of Fruit Tree Viruses 

HTS is now entering in the routine detection of plant viruses, either in cases of unknown etiology or in quarantine analysis of plant material trades. Facilitated by the decrease of costs, HTS is going to become the method of choice for virus discovery and identification in fruit trees. Scientific discussions are now dealing with the challenges of the technique and with the definition of laboratory standards for its application (see EPPO–COST FA1407 workshop, 2017; https://www.eppo.int/MEETINGS/2017_meetings/wk_ngs_diagnostics).

### 4.1. Identification of New Viruses in Fruit Tree Species with the Aid of High-Throughput Sequencing

The worldwide application of HTS to the analysis of plant viruses enlarges the potential of the technology, as revealed by the number of reports, in many economic relevant crops, as well as in eco-genomics studies that deal with the description of novel viruses. Here, we report a most updated list of them (Table 2), with the awareness that an almost daily increase of the viruses and virus-like pathogens here listed is going to deeply modify our knowledge of the “virus world” and its association with plant diseases.

(a) Citrus

The list of citrus virus-like diseases with unknown etiology was updated since 2012, with two publications on citrus yellow vein clearing of lemon trees [61] and citrus chlorotic dwarf [52]. In the first case, a new alphaflexivirus (*Citrus yellow vein clearing virus*, CYVCV) was discovered by siRNA analysis in Turkish accessions, and it was related to, but sufficiently divergent from the *Indian citrus ringspot virus* in the genus *Mandarivirus*. In the latter disease, a new viral DNA sequence was associated with the symptoms through siRNAs and total DNA sequencing. This new, highly divergent monopartite geminivirus was named *Citrus chlorotic dwarf associated virus* (CCDaV).

In order to identify the causal agent of citrus vein enation disease, Vives et al. [62] examined the siRNA fraction from infected and healthy Etrog citron plants. Contigs assembled from viral siRNAs showed similarity with luteovirus sequences, and determined the genome of Citrus vein enation virus (CVEV), related to the *Enamovirus* genus. 

A series of studies on citrus leprosis-associated viruses was described by Roy and co-authors. This disease, present in South and Central America and transmitted by mites, is caused by two differently shaped and taxonomically allocated viruses. Roy et al. [63,64] described, in Mexican leprosis-affected citrus plants, the sequence of two genomic components of a new rhabdo-like virus, similar to *Orchid fleck virus*, and attributed it to the proposed and still unassigned genus *Dichoravirus*. From Citrus leprosis symptomatic leaves from Colombia, Roy et al. [65] produced an siRNA library and found a novel cytoplasmic *Citrus leprosis virus* atypical strain (CiLV-C2; *Cilevirus*), plus *Citrus tristeza virus* (CTV) and citrus viroid sequences. This new virus produced symptoms typical of CiLV, but was not detected with either serological or PCR-based assays targeting the previously described cytoplasmic virus. Finally, the same group detected in Florida, on Carrizo citrange analyzed for citrus decline disease/citrus blight, three different endogenous pararetroviruses, significantly matching the *Petunia vein clearing virus* genome [66]; while, in a herbarium specimens in Florida, more than 50 years old, near full genome sequences of CiLV-N and -C species were recovered from the siRNA fraction [67].

A siRNAs library was prepared in Italy from orange trees affected by concave gum, a severe citrus disease reported more than 80 years ago and still with an unknown etiology. Navarro et al. [68] identified a new virus (with two genomic components of negative stranded RNA) similar to tenuiviruses and phleboviruses. This novel virus, tentatively named Citrus concave gum-associated virus, is flexuous and non-enveloped. While the viral agent associated to citrus sudden death disease, which appeared in Brazil in 1999, has been already characterized as belonging to a tymovirus-like species, and named as *Citrus sudden death-associated virus* (CSDaV), and an effort to further clarify the virome linked to disease expression was done by Matsumura et al. [69]. The sequencing analysis of the total RNA and siRNAs from CSD-symptomatic and -asymptomatic plants allowed the identification of both CSDaV and CTV in multiple virus infections, and the differentiation of viral genotypes. The still complicated virome picture identified in those plants consists of CTV as the most predominant virus (in an area in which the virus is endemic), followed by CSDaV, Citrus endogenous pararetrovirus (CitPRV), and two putative novel viruses tentatively named Citrus jingmen-like flavivirus and Citrus virga-like virus. The latter two viruses seem to be more associated with asymptomatic plants.

(b) Stone fruits

A pioneering work on HTS application to *Prunus* for de novo discovery and detection of viruses started from the INRA–Bordeaux team in 2013. Candresse et al. [43] analyzed a dsRNA library from sour cherry showing symptoms of Shirofugen stunt disease (SSD). The failure to detect any other viral agent, but a divergent isolate of *Little cherry virus-1* (LChV1), suggested that LChV1 could be responsible for the SSD syndrome.

From a cherry tree collected in Italy, and a plum tree collected in Azerbaijan, both with mixed-infections and no symptoms, Marais et al. [45] reconstructed contigs having weak but significant identity with various members of the family *Betaflexiviridae*. The identified viral species showed sequence homology with the sole member of the genus *Tepovirus*, *Potato virus T* (PVT). The name *Prunus virus T* was proposed for this new virus, which has 1% prevalence in the screening of a large *Prunus* collection.

Subsequently, during a field survey in apricot orchards, vein clearing and mottling symptoms were observed by Elbeaino et al. [44] on young leaves of a 3-year-old apricot tree. None of the main *Prunus* viruses were detected, while sequencing of dsRNA allowed the identification of a novel virus showing the highest nucleotide identity (ca. 40%) with *Citrus leaf blotch virus* (CLBV). The borderline phylogenetic allocation of this virus, namely Apricot vein clearing associated virus (AVCaV), between citri- and vitiviruses (presence of four distinct encoded proteins) led to a proposal for a new genus in *Betaflexiviridae*. Indeed, a further report from Marais et al. [70] identified two more isolates of AVCaV from almond and Japanese plums, and an additional new virus sequence in an Azerbaijani almond tree (Aze204) that was considered as a new distinct species (named *Caucasus prunus virus*, CPrV). The authors suggested that AVCaV and CPrV, having close genetic and structural similarity, could be integrated in the newly created genus *Prunevirus*. Anyway, while Elbeaino et al. [44] identified AVCaV in mixed-infections, the failure to detect any other viral agent in the Aze204 source indicated the putative involvement of CPrV in the etiology of the mild symptoms observed (chlorotic spots along the veins and weak reddening of young leaves).

Additional work was presented on the full genome sequencing of the Asian prunus viruses. Marais et al. [46] reconstructed the complete genomes of *Asian prunus virus 1*, *2*, and *3* (APV1, APV2, and APV3). A relevant phylogenetic assessment was that APV2 should be considered as a distinct viral species in the genus *Foveavirus*, while taxonomies of APV1 and APV3 were not yet defined. In the most recent report from this group [71], the new capillovirus Mume virus A was described in a Japanese apricot (*Prunus mume*) showing diffuse chlorotic spots on leaves. Lenz et al. [48], in Czech Republic, described in several mixed-infected cherry accessions (having no particular symptoms) a new virus sequence, named cherry-associated luteovirus (ChALV). This is the fourth member of the family *Luteoviridae* reported to naturally infect woody plants. After this discovery, sequence homologies to ChALV were identified by Wu et al. [72] in the United States in peach accessions from Spain and Georgia. A clade of *Rosaceae*-associated luteoviruses (rose spring dwarf, nectarine stem pitting, etc.) was therefore conceived and proposed. 

A sequencing project performed in California [73] on dsRNA extracts from imported nectarine accessions that showed stunting and pitting on woody cylinder in a propagation block, revealed the presence of a novel luteovirus (nectarine stem pitting-associated virus, NSPaV). Once again, the importance of metagenomic analysis in post-entry quarantine to assess plant health status is underlined. Villamor et al. [74] using total RNA instead of dsRNA on the same format (50 nucleotide single read Illumina run) sequenced, besides the NSPaV found in California, a new virus genome resembling a typical marafivirus (Nectarine virus M, NeVM) in symptomatic *P. persica* trees; the same marafivirus and luteovirus were found in symptomless nectarine and peach selections, which tested negative by indexing or free from known viruses by serological and molecular tests. In the attempts to clarify the complex association and genomic relationship of several virus-like diseases and the related agents in cherry, Villamor et al. [75] did a comprehensive analysis of previously reported full genomic sequences (*Cherry green ring mottle virus*, and *Cherry necrotic rusty mottle virus*) and determined new sequences from isolates of *Cherry twisted leaf-associated virus* (CTLaV) and *Cherry rusty mottle-associated virus* (CRMaV). Finally, the segregation of these viral sequences into four clades corresponding to distinct virus species was assessed. The establishment of a new genus (*Robigovirus*), gathering these viruses, was then suggested within the family *Betaflexiviridae*.

Villamor et al. [49] revealed a new virus resembling fabaviruses when they analyzed rRNA-depleted total RNA, extracted from a sweet cherry tree showing angular necrotic leaf spot symptoms, a general decline, and loss of fruit-bearing shoots. This virus (with a bipartite genome) was named *Prunus virus F* (PrVF), and it was found associated in the sample along with other common *Prunus*-infecting viruses. 

More new *Prunus* viruses were also identified during recent HTS screening and surveys in Asia. He et al. [38] sequenced the siRNAs from peach plants affected by a disease causing smaller and cracked fruit and searched for viral pathogens. Besides already known viruses, a novel fabavirus, namely Peach leaf pitting-associated virus (PLPaV) was identified, having some genome differences in the genome features from other members of the genus. More recently, one more *Fabavirus* species, CVF, was identified by Koloniuk et al. [40] infecting sweet and sour cherry trees in Greece and Czech Republic. CVF is closely related to PrVF, although the two viruses may differ in the expression strategy of their RNA2 genomic segments. 

Igori and co-workers described the complete sequence of a highly divergent South Korean isolate of a ChALV from peach [76]. Later on, from leaves exhibiting symptoms of yellowing and slight mottling collected from a yard-grown peach tree, they assembled the complete genome of the novel Peach virus D (PeVD) [77]. Sequence comparisons and phylogenetic analysis revealed that PeVD is most closely related to viruses in the genus *Marafivirus*, with a closer relationship (56–61% amino acid identity) with Nectarine virus M (NeVM), recently reported to infect nectarine. Similarly, Jo et al. [78] describing the virome of six peach trees, again in South Korea, by metatranscriptome analysis, identified five known betaflexiviruses, and a novel virus belonging to the family *Tymoviridae*, having a very close similarity to PeVD.

(c) Pome fruits

HTS analysis was applied to study the occurrence of symptoms of small leaves and growth retardation in field-grown apple trees in Korea [79]. The study showed that plants were multiply infected by at least two of the following viruses: ACLSV, *Apple stem grooving virus* (ASGV), *Apple stem pitting virus* (ASPV), Apple green crinkle associated virus (AGCaV), and *Apricot latent virus* (ApLV). HTS analysis of an siRNA library allowed for the discovery of an isolate of AGCaV in field-grown quince plants showing symptoms of fruit deformation and bud failure [80]. In a successive survey, the virus was found consistently associated with symptoms.

An apple geminivirus was discovered in apple by sequencing an siRNA library [34]. The virus had a limited prevalence in Chinese cultivars without association with specific symptoms. Moreover, a comprehensive HTS study of the virome of apple trees with symptoms of mosaic not associated with *Apple mosaic virus* (ApMV), led to the discovery of a novel ilarvirus, closely correlated with ApMV, which was named Apple necrotic mosaic virus (ApNMV) [81].

A couple of publications deal with the study of Apple rubbery wood disease by HTS analysis. While Jakovljevic et al. [82] failed in identifying putatively associated virus(es) by sequencing ribosomal RNA (rRNA)-depleted total RNA from infected plants, other authors [83] enlarged the previous dataset with the analysis of additional dsRNA libraries. A complex virome, containing two new viral species, Apple rubbery wood virus 1 and 2 (ARWV 1 and ARWV 2), allowed for establishment of a strong link among Apple rubbery wood disease and the Apple rubbery viruses. ARWVs have a multi-segmented negative sense RNA genome and represent unique members of the *Bunyavirales*. The authors suggest a taxon as a new genus under the *Phenuiviridae* family. Finally, Apple-associated luteovirus (AaLV) is a new species in the genus *Luteovirus* discovered by HTS sequencing of a library of rRNA-depleted total RNAs [84]. AaLV was found in asymptomatic apple plants of different cultivars in China and, as such, is considered a latent virus.

(d) Examples of High-Throughput Sequencing based virus discovery on minor fruit trees (persimmon, mulberry, actinidia)

A short survey on the literature regarding the minor fruit tree species can be presented to further support the wide potential of HTS discovery when applied to some “neglected” virus-like plant disorders. 

Ito et al. [85] detected two genomes of graft transmissible viruses in total RNA extracts (treated with S1 nuclease to enrich for dsRNA) of Japanese persimmon trees exhibiting fruit apex disorder. One of the complete genomes consisted of 13,467 nucleotides and encoded genes similar to those of plant cytorhabdoviruses (named Persimmon virus A, PeVA). The other genome shared an organization similar to those of some insect and fungal viruses having dsRNA genomes. The virus, named Persimmon latent virus (PeLV), formed a possible new genus clustering with two insect viruses and one plant virus, Cucurbit yellows-associated virus. Later on, two dsRNA molecules of about 1.5 kb in size were sequenced by Morelli et al. [86] from leaf tissue, showing veinlets necrosis, of a Japanese persimmon from Southern Italy. The identified virus, Persimmon cryptic virus (PeCV), which is close to deltapartitiviruses, was, however, also found in symptomless persimmon trees and seedlings.

In mulberry, a library from siRNAs allowed Ma et al. [87] to identify a novel DNA virus (*Mulberry mosaic dwarf associated virus*) in a Chinese accession affected by mosaic and dwarfing symptoms. This virus has a small monopartite circular DNA genome (2.95 kb) containing ORFs (open reading frames) in both polarity strands, resembling CCDaV, a divergent geminivirus recently identified in citrus (see Loconsole et al. [52]). The *Mulberry badnavirus 1* (MBV1) has been characterized as the etiological agent of a disease observed on a mulberry tree in Lebanon by Chiumenti et al. [35]. An siRNA library was analyzed, and these data, together with genome walking experiments, generated the full-length virus sequence. Uniquely among badnaviruses, the MBV1 sequence encodes a single ORF containing all the conserved pararetrovirus motifs, while a defective genome was found to replicate and be encapsidated in infected plants.

Wang et al. [88] sequenced siRNAs from kiwi fruit (*Actinidia* spp.) in China, and provided the first complete genome sequence of an ASGV isolate (ASGV-Ac) infecting a kiwi fruit plant. This sequence is phylogenetically distant to ASGV isolates from all other hosts by sharing about 80% genome sequence identity, and likely represents a novel variant. Zheng et al. [89] characterized a new virus infecting kiwi fruit vines affected by chlorotic ringspots. The virus (Actinidia chlorotic ringspot-associated virus, AcCRaV), presents double-membrane bodies in infected tissues, and has a genome composed of RNA segments in negative polarity, so it covers the typical features of members in the genus *Emaravirus*. A final report from New Zealand [90] described the complete genome sequence of a novel virus, tentatively named Actinidia seed-borne latent virus (ASbLV). The virus was identified from *Actinidia chinensis* (a seedling in home garden), its genome contains four open reading frames, and is most closely related to CPrV (56% nt identity), a member of the genus *Prunevirus* in *Betaflexiviridae*. Plants were affected by vein chlorosis and mottling, but were found to be co-infected with *Actinidia virus A* and/or Actinidia virus 1, although ASbLV was also detected in asymptomatic vines as a single infection.

### 4.2. Application of High-Throughput Sequencing to Detection and Virus Genetic Variability Studies

The increasing use of HTS techniques for a rapid and sensitive detection of the virome in particular plant material (virus source collections, plant clinics, mother plants for certified stocks, etc.) is essentially devoted to have a complete picture of the systemic agents (for what is known in that host). This application is generally intended as “targeted”, when generic or even specific primers are able to amplify all the variability in a particular target sequence and compare, simultaneously, up to several hundreds of samples in a single run. Alternatively, the unbiased, or “untargeted”, sequencing of many samples can also aim to describe the fine-tuning of nucleotide variability in the quasi-species frame. A by-product of this analysis is the refinement of conventional molecular diagnostic tools through the additional genome variability information gained.

At least two applications for a massive genotyping and virus characterization through HTS can be reported. The first one is the screening for dominant strains or isolates in endemic areas where cross-protection against dangerous pathogens is performed. In Brazil or South Africa, mild CTV strains are used to control the emergence of severe strains. In these cases, accurate monitoring of newly emergent strains, that can occur by introduction or recombination, can be used for timely containment actions. A second application is an eco-genomic survey in certain crops or for certain virus groups, that is, run necessarily as a pilot study in geographic areas (see the example of Australia) [91] to check the virus status already present, and avoid the introduction of unwanted agents. Pursuing the former objective, Zablocki & Pietersen [92] and Read & Pietersen [93] provided evidence through HTS analysis regarding the isolates associated with the breaking of CTV cross-protection in grapefruit industry, characterizing recombination events, and fine population mapping, even at a single gene level. An example of the study of fine molecular virus–host interactions is the report of Visser et al. [94] who sequenced siRNAs and transcripts on grapefruit co-infected by CTV and *Citrus dwarfing viroid*. Analyzed siRNAs showed the pathogen regions associated with increased silencing and an altered plant hormone regulatory pattern. The same group developed and evaluated e-probes to be applied on citrus massive sequencing for rapid, simultaneous identification of 11 recognized viruses and viroids [95]. This e-probe-based approach was applied to screen different samples and RNA template, and mainly for CTV genotyping.

In the frame of CTV research, Yokomi et al. [17] sequenced siRNAs of Californian CTV isolates with divergent serological and molecular profiles and trifoliate resistance-breaking isolates were identified, while Licciardello et al. [96] analyzed siRNAs from two CTV isolates (aggressive and mild), representing the population structure of the virus in Sicily. Their molecular data needed, however, to be integrated with bio-indexing, in order to obtain adequate information for risk assessment. The genome of a one-century old lemon-infecting CTV isolate was reconstructed in Greece through siRNAs sequencing on Ion Torrent platform (Table 3). The isolate, even if it belongs to a severe strain, shows mild symptoms and bears signal of recombination events.

Other records of variability of citrus viruses were done in Japan [97], where five isolates of CVEV were determined by total RNA sequencing and compared, and in China [98], with the siRNA detection of a Chinese isolate of CYVCV.

On the side of stone fruit, more attention was paid to date to some less characterized viruses such as LChV1, *Plum bark necrosis stem pitting-associated virus* (PBNSPaV) or *Cherry virus A* (CVA), as well as on newly identified fabaviruses. Studies on ilarviruses in Australian stone fruits focused on genetic variability of a number of isolates obtained by generic amplicon sequencing [99]. 

More specifically, several variants of PBNSPaV were retrieved from a project of sequencing four *Prunus* samples of unclear or unknown etiology [100], while a first isolate from sweet cherry was sequenced in China [101]. Recently, isolates of LChV1 were sequenced from peach in South Korea [102], apricot in Hungary [41], and sweet cherry in China [103]. Full genome sequences from isolates of CVA were obtained from apricot in Japan [104] and Hungary [41], and from sweet cherry in China [105]. In a wild cherry tree from Slovakia, together with a multiple viral infection, Glasa et al. [9] found two divergent isolates of CVA (differing by 14% at the nucleotide level) and gave some evidence on the recombinant nature of these isolates and for the presence of “non-cherry” group CVA isolates in cherry host plants. CVA variability was studied also in Canada by Kesanakurti et al. [50] in 39 stone fruit tree specimens infected with CVA, in which 75 full and 16 partial-length CVA genomes were assembled. No phylogenetic relationship was found between the host and the assembled genome variants.

Using sequencing of dsRNA on PCR positive samples Špak et al. [106] obtained nearly complete genomes of one CNRMV and three CGRMV isolates, concluding, by their genetic relationship with foreign isolates and negative results screening in a plant collection for these viruses, that their origin was from imported accessions.

Three publications from a project on *Ilarvirus* species in Australia interestingly applied a strategy of sequencing of double-indexed amplicons. In a first study [107], the variability of *Prunus necrotic ringspot virus* (PNRSV) was investigated on 53 PNRSV-infected trees in conserved gene regions of each of PNRSV RNA1, RNA2, and RNA3. Some plant samples had sequence variants occurring in multiple PNRSV phylo-groups, and a nucleotide identity of 97% was determined as demarcation threshold for each of the PNRSV phylo-groups, each one composed by distinct clades of polymorphic variants. A second study [99] addressed, through generic amplicon sequencing (in RNA2), the distribution of Ilarvirus species populations amongst 61 Australian *Prunus* trees from a wide survey in stone fruit orchards. Mixed-infections of ilarviruses were detected, and also two different RNA2 sequence variants most likely belonging to unknown ilarviruses, from which no other genomic RNAs were identified. In a survey of 127 *Prunus* tree samples collected from five states in Australia, Kinoti et al. [108] found ApMV and PDV to occur in 3% and 10% of the trees, respectively. HTS of amplicons from partial conserved regions of RNA1, RNA2, and RNA3 of ApMV and PDV was used to determine the genetic diversity of the Australian isolates of each virus.

Furthermore, by using HTS, Koloniuk et al. [40] described the co-infection of sweet and sour cherry trees with diverse genomic variants of two closely related fabaviruses, namely PrVF and CVF. A similar observation was done by Villamor et al. [49] during the initial identification of PrVF. It is particularly interesting that arbitrary mixtures of phylogenetically distinct genotypes of the two genomic segments from both viruses exist in the sampled trees, while the occurrence of single genotype infections is rare. 

Finally, in HTS research of virus variability in apple, Visser et al. [33] analyzed siRNA libraries from plants infected by ASGV. The analysis of data demonstrates that, in apple, the anti-viral RNA silencing operates similarly to other plant species by producing predominant 21–22 nucleotide-long viral siRNAs, and these originate mainly from the 3′ end of the viral genome, where subgenomic RNAs are synthesized. In addition, publicly available mRNA and siRNA libraries from apple were mined by Jo et al. [57] to survey for the presence of ASGV and obtain information on genome recombination, single nucleotide variants, and phylogenetic relationships. The work illustrates the suitability of already available datasets to be used in successive investigations and for multiple purposes. Table 3 summarizes detection and fruit tree virus genetic variability studies applying HTS. 

## 5. Advantages and Disadvantages of High-Throughput Sequencing Compared to Classical Diagnostic Approaches

The application of HTS technologies has several advantages over the classical serological, molecular, and biological approaches both for disease diagnosis and for specific virus detection in fruit trees. Among the most important advantages of HTS is that a priori knowledge on the viral pathogens infecting a plant sample is not required. This means that there is no need to know the sequence of a targeted virus species, as in PCR, in order to design primers for its molecular detection or, like ELISA, to have antibodies available for serological detection. The classical detection assays, with the exception of biological indexing, are highly specific and cannot identify unknown viruses that might be involved in the etiology of a plant disease. The use of HTS reduces the time needed, and facilitates the necessary procedures in order to identify the etiological agent of a disease, since the method provides the identification of the plant sample’s virome, often unveiling the presence of novel viral pathogens [38,49,53,74,111,112]. 

As far as virus detection is concerned, the application of deep sequencing approaches alleviates the problem of false negatives due to high genetic variability or low titer, which are frequently encountered in viruses infecting fruit trees [113,114]. Moreover, the high number of different reads obtained from the HTS run makes possible the reconstruction of the full genome sequences of a virus species, which can be subsequently used to perform genetic variability studies. Complete viral genomes or big fragments of them have been obtained using either siRNAs [33,34,35,36,37,38,39,40,41], dsRNA [40,43,44,45,46,48,49,50,71,106], or total RNA [9,40,49,57,76] template from a multitude of different fruit tree plants. Where HTS excels, however, is the identification of genetic variants of a virus species that often co-exist in the same perennial host. The ability to produce full genomes from *de novo* assembly, in combination to sequence depths which can reach up to a few thousand reads per site, can reveal the presence of variants in very low frequencies, as well as defective RNAs or mixed-infections of closely related strains/isolates [9,40,49]. It should be noted, however, that validation of the sequences obtained by HTS can be needful, for which the conventional Sanger sequencing is applied. This verification usually concerns poorly covered genome portions, the 5´and 3´genome extremities or the integrity of unusual recombination junction site(s).

In woody perennial crops, mixed virus infection is the most common feature, so unravelling diseases of unknown etiology is complicated by the simultaneous presence of different viruses (potentially acting in synergism/antagonism), and the presence/absence of a novel key virus should be further confirmed by conventional detection in wider surveys. Symptoms can be linked to peculiar, severe isolates of a virus species, thus the recovery of specific sequences maybe not enough for causality association. In the citrus sudden death disease [69], a specific strain of CSDaV seems to be associated with the expression of symptoms, and several other viruses with higher abundance of reads are present in symptomless plants, along with different strains of CSDaV. Another case is the discovery of an atypical cytoplasmic citrus leprosis virus strain (CiLV-C2) [63], found by HTS and not detected by molecular tools specific for CiLV-C. On the other hand, HTS often produces sequences belonging to non-plant pathogenic agents, such as mycoviruses, which were identified in grapevine [53], or pararetrovirus sequences that possibly represent genetic fossils integrated in the plant genome [115]. In addition, HTS has revealed, over the last few years, the presence of a large number of new virus species, the biological significance of which is unknown. For some of those viruses, a number of biological traits can be inferred based on the homology they share with already characterized species, however, for many, it is even still debatable whether they represent plant-infecting viruses having economic importance [85,116,117]. The identification of a new plant virus species could affect trading of plant material across countries, and therefore, biological data should be provided soon after the initial identification of the pathogen, in order to better assess its significance [118]. 

Despite the advances in the field of HTS technology and the ability to sequence several samples simultaneously as a pool of extracted nucleic acid, testing the large number of samples necessary to study the incidence or molecular epidemiology of a plant virus species is still costly. In addition, there are no established thresholds of reads numbers to identify the presence of a virus species in a sample, which needs to assess the sanitary status of a sample. The detection of a low number of reads from a viral genome could indicate either the presence of a virus in low titer, or the contamination of the sample during handling. Therefore, the additional application of species-specific molecular/serological techniques is still needed in order to confirm such findings. Another drawback of high-throughput technologies is the impact of the selected template on the kind of viral pathogens detected. This template-dependency might be observed for DNA viruses which are excluded from the analysis of dsRNA-based libraries and, likewise, after poly(A) enrichment, where viruses lacking a poly(A) tail are not detected. On the other hand, VANA has been reported to successfully detect both RNA and DNA viruses [119,120] but it is not able to detect agents that are not encapsidated, such as viroids and endornaviruses. Given each approach’s benefits and limitations, serious consideration should be given to the selection of the template used. Τhe majority of tree-infecting viruses have RNA genomes, have a dsRNA stage during their replication and might have a poly(A)tail and get encapsidated for transmission and movement. However, the recent rise in identification of tree-infecting DNA viruses [52,121,122], viruses lacking poly(A) tail (e.g., luteoviruses) [48,72,74,76], and the importance of viroid-related diseases possibly make siRNAs or total RNA the templates of choice for a thorough identification of the viruses present in a sample. An important drawback of HTS is the fact that, contrary to the classical detection methods, the analysis of HTS data requires the use of sophisticated bioinformatics tools by specialized scientific personnel. Depending on the template analyzed, different obstacles might be encountered during the bioinformatic analysis. It is probably necessary to further optimize the pipeline used for the analysis e.g., in RNAseq analysis, a step of subtracting the plant genome should be included in order to easily identify new (and known) viruses, since the majority of the resulting contigs are plant-derived. Otherwise, when the plant species genome is not available, it is a highly laborious task to identify the contigs related to new viruses from the thousands of contigs produced from the de novo assembly. 

Finally, it should be highlighted that diagnostic interpretation of the bioinformatic analysis of HTS data is of paramount importance compared to interpretation of the results of classical detection assays. Whichever the template used for the analysis, it is highly probable that a multitude of different viruses will be found in a sample. Perennial host trees accumulate viruses over the years and those might, or might not, play a role on the development of a disease. The presence of a virus in a diseased sample is not proof of the induction of the observed symptomatology and caution should be even taken when naming a new virus, as the inclusion of the disease in the virus name could predispose the research on this virus, and might have implications on the movement of propagative material across borders. Characterization of a newly identified virus and its role in disease etiology would probably require the use of infectious complementary DNA clones or other biological techniques, enabling establishment of a single agent infection of a plant [118].

## 6. Future Directions of High-Throughput Sequencing in Fruit Tree Virology

HTS analysis is undoubtedly a technological breakthrough that has yet to offer a lot in plant virology. However, advances in the field of fruit-tree virology using HTS have to mitigate the weaknesses of the other assays, mainly those related to pathogen diagnosis and the identification of the sanitary status of the trees. Those two aspects of research are directly involved in the production of virus-tested high-quality propagative material, and the prevention of trading of diseased plants which are the main ways of containment for serious fruit tree viral diseases. The perennial life cycle of tree crops places virus infections into a different perspective than those of the annual herbaceous plants. In the latter, the diseases and viral loads might change each year, and annual fluctuations are observed even in areas where specific viruses are endemic. In fruit trees, however, viruses accumulate over the years, giving rise to mixed-infections, which become the normal status of a tree. Moreover, the initial sanitary status of each plant from the nursery might play a major role on the development of a disease through the interactions of the viruses present. Therefore, it will be highly important to further improve current HTS technologies and subsequent bioinformatic analysis procedures, so that they can be efficiently applied in the fields of certification of fruit tree propagation material and phytosanitary control.

HTS will eventually lead to the identification of the majority, if not every, virus and viroid present in each host species, updating their virome data. This extensive database, of different isolates from the viruses infecting trees, will be able to optimize the existing detection assays by addressing one of the major shortcomings for the traditional methodologies, which is the high sequence variability present in fruit tree-infecting viruses. The optimization of the existing primers based on sequences from different isolates, and the variants of these isolates present in a sample, will minimize false-negative results, thus leading to more accurate diagnostic assays. Moreover, the assessment of the new viruses identified will provide a thorough list of pathogens posing potential threats, and quarantine lists can be updated.

HTS has proven a valuable tool in the identification of genotype mixtures in the form of virus variants, and different isolates inside a single plant. With the aid of this technology, the study of interactions between different viruses or virus genotypes will be facilitated, unveiling mechanisms of pathogenesis and disease development. Importantly, the method could be used for the study of plant–virus–vector interactions through transcriptomic analyses or studies of resistance mechanisms, such as RNA silencing, acting against viruses in different fruit tree species, which in the end, could lead to the development of novel control tools.

## Figures and Tables

**Table 1:** Currently commercially available high-throughput sequencing equipment.

Platform		Maximum Read Length (bp)	Sequencing Output	Maximum Number of Reads per Run	Run Time
**PCR based HTS**					
Illumina	iSeq 100 System	2 × 150	1.2 Gb	4 million	9–17.5 h
	MiniSeq System	2 × 150	7.5 Gb	25 million	4–24 h
	MiSeq System	2 × 300	15 Gb	25 million	4–55 h
	NextSeq 550 System	2 × 150	120 Gb	400 million	12–30 h
	HiSeq 2500 System	2 × 125/2 × 250	1000Gb/300 Gb	4 billion/600 million	29 h–6 days/7–60 h
	HiSeq 3000 System	2 × 150	750 Gb	2.5 billion	<1–3.5 days
	HiSeq 4000 System	2 × 150	1500 Gb	5 billion	<1–3.5 days
	HiSeq X Series	2 × 150	1800 Gb	6 billion	<3 days
	NovaSeq 6000 System	2 × 150	6000 Gb	20 billion	16–44 h
Ion Torrent	PGM (314 chip)	200/400	50/100 Mb	550 thousand	2.3/3.7 hr
	PGM (316 chip)	200/400	300 Mb/1 Gb	3 million	3.0/4.9 hr
	PGM (318 chip)	200/400	1/2 Gb	5.5 million	4.4/7.3 h
	Proton PI chip	200	10 Gb	80 million	2–4 h
	Ion Gene Studio S5 (510 chip)	200/400	0.5/1 Gb	3 million	4.5/10.5
	Ion Gene Studio S5 (520 chip)	200/400/600	1/2/1.5 Gb	6/6/4 million	7.5/12/12 h
	Ion Gene Studio S5 (530 chip)	200/400/600	4/8/4.5 Gb	20/20/12 million	10.5/21.5/21 h
	Ion Gene Studio S5 (540 chip)	200	15 Gb	60 million	19 h
	Ion Gene Studio S5 Plus (550 chip)	200	25 Gb	100 million	11.5 h
**SMS NGS**					
Pacific Biosciences	Sequel System	>1000	1–10 Gb	500.000	0.5–10 h
	PacBioRS II	>1000 (average 10.000) up to 60.000	1–10 Gb	1–10 million	0.5–6 h
Oxford Nanopore	MinION	5000 to >100.000	10–20 Gb	-	48 h
	GridIONx5	5000 to >100.000	50–100 Gb	-	48 h
	PromethION	5000 to >100.000	1.2 Tb	-	48 h

PGM: Personal Genome Machine.

**Table 2:** De novo discoveries.

Virus	Genus	Family	Host	Reference
Actinidia chlorotic ringspot-associated virus	*Emaravirus*	*Fimoviridae*	Actinidia	Zheng et al. [89]
Actinidia seed-borne latent virus	*Prunevirus*	*Betaflexiviridae*		Veerakone et al. [90]
Apple-associated luteovirus	*Luteovirus*	*Luteoviridae*	apple	Shen et al. [84]
Apple geminivirus	*Geminivirus*	*Geminiviridae*		Liang et al. [34]
Apple necrotic mosaic virus	*Ilarvirus*	*Bromoviridae*		Liang et al. [34]
Apple rubbery wood virus 1	(?)	*Phenuiviridae*		Rott et al. [83]
Apple rubbery wood virus 2	(?)	*Phenuiviridae*		Rott et al. [83]
Citrus concave gum-associated virus	*Phlebovirus*(?)	*Phenuiviridae*	Citrus	Navarro et al. [68]
Citrus jingmen-like virus	*Flavivirus*	*Flaviviridae*		Matsumura et al. [69]
Citrus leprosis virus-N	*Dichoravirus*	*Rhabdoviridae*		Roy et al. [65]
Citrus leprosis virus-C2	*Cilevirus*			Roy et al. [63]
*Citrus yellow vein clearing virus*	*Mandarivirus*	*Alphaflexiviridae*		Loconsole et al. [61]
Citrus vein enation virus	*Enamovirus*	*Luteoviridae*		Vives et al. [62]
Citrus virga-like virus	*Virgavirus*	*Virgaviridae*		Matsumura et al. [69]
Citrus chlorotic dwarf-associated virus	*Geminivirus*	*Geminiviridae*		Loconsole et al. [52]
*Cherry Rusty Mottle-associated virus*	*Robigovirus*	*Betaflexiviridae*	cherry	Villamor et al. [75]
*Cherry Twisted Leaf-associated virus*	*Robigovirus*	*Betaflexiviridae*		Villamor et al. [75]
*Prunus virus F*	*Fabavirus*	*Secoviridae*		Villamor et al. [49]
Cherry virus F	*Fabavirus*	*Secoviridae*	sweet cherry, sour cherry	Koloniuk et al. [40]
Mulberry mosaic dwarf-associated virus	(?)	*Geminiviridae*	mulberry	Ma et al. [87]
Mulberry badnavirus 1	*Badnavirus*	*Caulimoviridae*	mulberry	Chiumenti et al. [35]
Nectarine stem pitting-associated virus	*Luteovirus*	*Luteoviridae*	peach	Bag et al. [73]
Nectarine virus M	*Marafivirus*	*Tymoviridae*		Villamor et al. [74]
Peach leaf pitting-associated virus	*Fabavirus*	*Secoviridae*		He et al. [38]
Peach virus D	*Marafivirus*	*Tymoviridae*		Igori et al. [77]
Persimmon cryptic virus	*Deltapartitivirus*	*Partitiviridae*	persimmon	Morelli et al. [86]
Persimmon virus A	*Cytorhabdovirus*	*Rhabdoviridae*		Ito et al. [85]
*Apricot vein clearing associated virus*	*Prunevirus*	*Betaflexiviridae*	Prunus	Elbeaino et al. [44]
*Caucasus prunus virus*	*Prunevirus*	*Betaflexiviridae*		Marais et al. [70]
Cherry-associated luteovirus	*Luteovirus*	*Luteoviridae*		Lenz et al. [48]
Mume virus A	*Capillovirus*	*Betaflexiviridae*		Marais et al. [71]
*Prunus virus T*	*Tepovirus*	*Betaflexiviridae*		Marais et al. [45]

?: putatively belongs to a new virus genus.

**Table 3:** Detection and virus genetic variability studies.

Virus	Genus	Family	Host	Aim of the research & New Findings	Reference
*Apple stem grooving virus*	*Capillovirus*	*Betaflexiviridae*	apple	Differential expression of siRNAs in symptomless infections	Visser et al. [33]
*Apple stem grooving virus*	*Capillovirus*	*Betaflexiviridae*	Actinidia	First HTS-obtained ASGV genome in kiwi	Wang et al. [88]
*Asian prunus virus 1*	*Foveavirus*	*Betaflexiviridae*	Prunus	Genome reconstruction of Asian prunus viruses from 5 samples: phylogenetic and recombination implications	Marais et al. [46]
*Asian prunus virus 2*	*Foveavirus*	*Betaflexiviridae*			Marais et al. [46]
Asian prunus virus 3	*Foveavirus*	*Betaflexiviridae*			Marais et al. [46]
cherry-associated luteovirus	*Luteovirus*	*Luteoviridae*	peach	Highly divergent isolate in a new host (peach)	Igori et al. [76]
*Cherry necrotic rusty mottle virus*	*Robigovirus*	*Betaflexiviridae*	cherry	One CNRMV and three CGRMV isolates sequenced from a collection survey	Špak et al. [106]
*Cherry green ring mottle virus*	*Robigovirus*	*Betaflexiviridae*			
*Cherry virus A*	*Capillovirus *	*Betaflexiviridae*	apricot	First complete sequence by HTS (apricot isolate); Description in apricot on propagation material	Koinuma et al. [104]; Barath et al. [41]
*Cherry virus A*	*Capillovirus *	*Betaflexiviridae*	cherry	New isolate from China	Wang et al. [105]
*Cherry virus A*	*Capillovirus *	*Betaflexiviridae*		Coinfection of two divergent variants in a wild cherry tree	Glasa et al. [9]
*Cherry virus A*	*Capillovirus *	*Betaflexiviridae*		HTS survey on 39 Prunus samples: 75 full genomes and phylogenetic implications	Kesanakurti et al. [50]
Cherry virus F	*Fabavirus*	*Secoviridae*	sweet cherry, sour cherry	Virome of 9 samples: co-infection of different viruses and co-existence of multiple fabavirus variants	Koloniuk et al. [40]
Citrus sudden death virus	*Marafivirus*	*Tymoviridae*	Citrus	Virome analysis of CSD-affected plants: wide variability of genotypes and mixed infections with different viruses	Matsumura et al. [69]
*Citrus tristeza virus*	*Closterovirus*	*Closteroviridae*		Genotyping 3 cross-protecting CTV strains in grapefruit; CTV p33 amplicons sequencing on 92 field samples: high diversity between and within samples	Zablocki & Pietersen [92]; Read & Pietersen [93]
*Citrus tristeza virus*	*Closterovirus*	*Closteroviridae*		siRNAs study: altered siRNAs pattern in CTV-infected plants, hot spot on CTV genome	Visser et al. [94]
*Citrus tristeza virus*	*Closterovirus*	*Closteroviridae*		siRNAs analysis: mixed CTV genotypes in resistance-breaking isolates	Yokomi et al. [17]
*Citrus tristeza virus*	*Closterovirus*	*Closteroviridae*		Phylogenetic analysis on 2 CTV populations on 225 samples for epidemiology and risk assessment	Licciardello et al. [96]
*Citrus tristeza virus*	*Closterovirus*	*Closteroviridae*		siRNAs sequencing of an old lemon isolate of CTV	Varveri et al. [109]
*Citrus Yellow vein clearing virus*	*Mandarivirus*	*Alphaflexiviridae*		siRNAs analysis and phylogeny of 10 isolates of CYVCV	Yu et al. [98]
Citrus vein enation virus	*Enamovirus*	*Luteoviridae*		Characterization of 5 Japanese genomes of Citrus vein enation virus	Nakazono-Nakaoga et al. [97]
*Little cherry virus 1*	*Velarivirus*	*Closteroviridae*	peach	A new isolate sequenced from South Korea in alternate host (peach)	Lim et al. [102];
*Little cherry virus 1*	*Velarivirus*	*Closteroviridae*	sweet cherry	New isolate in China	Wang et al. [103]
*Little cherry virus 1*	*Velarivirus*	*Closteroviridae*	cherry	Genetic diversity among isolates and co-infection of different variants	Katsiani et al. [110]
*Little cherry virus 1*	*Velarivirus*	*Closteroviridae*	Prunus	First description in Hungary on apricot and in Spain on sweet cherry	Baràth et al. [41]; Ruiz-Garcia et al. [36]
*Plum bark necrosis stem pitting-associated virus*	*Ampelovirus*	*Closteroviridae*	Prunus	4 full genomes sequenced from Prunus sources	Marais et al. [100]
*Plum bark necrosis stem pitting-associated virus*	*Ampelovirus*	*Closteroviridae*	cherry	One complete genome from sweet cherry	Wang et al. [101]
*Prunus necrotic ringspot virus*	*Ilarvirus *	*Bromoviridae*	Prunus	Ilarvirus RNA2 generic amplicons from 61 trees: new potential ilarviruses; 53 PNRSV-infected sources: genetic strains network designed; incidence and genotyping of Australian ApMV and PDV	Kinoti et al. [99]; Kinoti et al. [107]; Kinoti et al. [108]
*Prune dwarf virus* & *Apple mosaic virus*	*Ilarvirus*	*Bromoviridae*			
*Prunus virus F*	*Fabavirus*	*Secoviridae*	sweet cherry, sour cherry	Virome of 9 samples: co-infection of different viruses and co-existence of multiple fabavirus variants	Koloniuk et al. [40]

HTS: High-throughput sequencing; ASGV: Apple stem grooving virus; CNMRV: Cherry necrotic rusty mottle virus; CSD: Citrus sudden death; CTV: Citrus tristeza virus; CYVCV: Citrus yellow vein clearing virus; PNRSV: Prunus necrotic ringspot virus; ApMV: Apple mosaic virus; PDV: Prune dwarf virus.

## References

[1] Hadidi A., Barba M., Hadidi A., Barba M., Candresse T., Jelkmann W. (2011). Economic impact of pome and stone fruit viruses and viroids. Virus and Virus-Like Diseases of Pome and Stone Fruits.

[2] Rubio M., Martínez-Gómez P., Marais A., Sánchez-Navarro J., Pallás V., Candresse T. (2017). Recent advances and prospects in *Prunus* virology. Ann. Appl. Biol..

[3] Nemeth M. (1986). Virus, Mycoplasma and Rickettsia Diseases of Fruit Trees.

[4] Varveri C., Holeva R., Bem F. (1997). Effect of sampling time and plant part on the detection of two viruses in apricot and one in almond by ELISA. Ann. BPI.

[5] Dal Zotto A., Nome S.F., Di Rienzo J.A., Docampo D.M. (1999). Fluctuations of *Prunus necrotic ringspot virus* (PNRSV) at various phenological stages in peach cultivars. Plant Dis..

[6] Jridi C., Martin J.F., Marie-Jeanne V., Labonne G., Blanc S. (2006). Distinct viral populations differentiate and evolve independently in a single perennial host plant. J. Virol..

[7] Ruiz-Ruiz S., Moreno P., Guerri J., Ambrós S. (2007). A real-time RT-PCR assay for detection and absolute quantitation of *Citrus tristeza virus* in different plant tissues. J. Virol. Methods.

[8] Predajňa L., Šubr Z., Candresse T., Glasa M. (2012). Evaluation of the genetic diversity of *Plum pox virus* in a single plum tree. Virus Res..

[9] Glasa M., Šoltys K., Vozárová Z., Predajňa L., Sihelská N., Šubr Z., Candresse T. (2017). High intra-host cherry virus a population heterogeneity in cherry trees in Slovakia. J. Plant. Pathol..

[10] Wood G.A. (1989). A system for propagating and virus-screening imported pome fruit and stone fruit cultivars under cross-hemisphere conditions. N. Z. J. Crop. Hortic. Sci..

[11] Jelkmann W. (2004). Detection of virus and virus-like diseases of fruit trees. Laboratory assays, bioassays and indicators. Acta Hortic..

[12] Damsteegt V.D., Stone A.L., Mink G.I., Howell W.E., Waterworth H.E., Levy L. (1998). The versatility of *Prunus tomentosa* as a biondicator of viruses. Acta Hortic..

[13] Gentit P. (2006). Detection of *Plum pox virus*: Biological methods. EPPO Bull..

[14] Matic S., Minafra A., Sánchez-Navarro J.A., Pallás V., Myrta A., Martelli G.P. (2009). ‘Kwanzan Stunting’ syndrome: Detection and molecular characterization of an Italian isolate of *Little Cherry Virus 1*. Virus Res..

[15] Legrand P. (2015). Biological assays for plant viruses and other graft-transmissible pathogens diagnoses: A review. EPPO Bull..

[16] Woo E.N.Y., Pearson M.N. (2014). Biological and molecular variation of Cherry leaf roll virus isolates from *Malus domestica*, *Ribes rubrum*, *Rubus idaeus*, *Rumex obtusifolius* and *Vaccinium Darrowii*. Plant Pathol..

[17] Yokomi R.K., Selvaraj V., Maheshwari Y., Saponari M., Giampetruzzi A., Chiumenti M., Hajeri S. (2017). Identification and characterization of *Citrus tristeza virus* isolates breaking resistance in trifoliate orange in California. Phytopathology.

[18] Glasa M., Palkovics L., Kominek P., Labonne G., Pittnerova S., Kudela O., Candresse T., Šubr Z. (2004). Geographically and temporally distant natural recombinant isolates of *Plum pox virus* (PPV) are genetically very similar and form a unique PPV subgroup. J. Gen. Virol..

[19] Torrance L., Jones R.A.C. (1981). Recent developments in serological methods suited for use in routine testing for plant viruses. Plant Pathol..

[20] Cambra M., Asensio M., Gorris M.T., Pérez E., Camarasa E., García J.A., Moya J.J., López-Abella D., Vela C., Sanz A. (1994). Detection of plum pox potyvirus using monoclonal antibodies to structural and non-structural proteins. EPPO Bull..

[21] Terrada E., Kerschbaumer R.J., Giunta G., Galeffi P., Himmler G., Cambra M. (2000). Fully “recombinant enzyme linked immunosorbent assays” using genetically engineered single- chain antibody fusion proteins for detection of *Citrus Tristeza Virus*. Phytopathology.

[22] López M.M., Bertolini E., Olmos A., Caruso P., Gorris M.T., Llop P., Penyalver R., Cambra M. (2003). Innovative tools for detection of plant pathogenic viruses and bacteria. Int. Microbiol..

[23] Hadersdorfer J., Neumuller M., Treutter D., Fischer T.C. (2011). Fast and reliable detection of *Plum pox virus* in woody host plants using the Blue LAMP protocol. Ann. Appl. Biol..

[24] Malandraki I., Beris D., Isaioglou I., Olmos A., Varveri C., Vassilakos N. (2017). Simultaneous detection of three pome fruit tree viruses by one-step multiplex quantitative RT-PCR. PLoS ONE.

[25] Lu Y., Yao B., Wang G., Hong N. (2018). The detection of ACLSV and ASPV in pear plants by RT-LAMP assays. J. Virol. Methods.

[26] Predajňa L., Sihelská N., Benediková D., Šoltys K., Candresse T., Glasa M. (2017). Molecular characterization of *Prune dwarf virus* cherry isolates from Slovakia shows their substantial variability and reveals recombination events in PDV RNA3. Eur. Plant Pathol..

[27] Glasa M. (2017). Personal communication.

[28] Radford A.D., Chapman D., Dixon L., Chantrey J., Darby A.C., Hall N. (2012). Application of next-generation sequencing technologies in virology. J. Gen. Virol..

[29] Margulies M., Egholm M., Altman W.E., Attiya S., Bader J.S., Bemben L.A., Berka J., Braverman M.S., Chen Y.J., Chen Z. (2005). Genome sequencing in microfabricated high-density picolitre reactors. Nature.

[30] Schendure J., Ji H. (2008). Next-generation DNA sequencing. Nat. Biotechnol..

[31] Kilianski A., Haas J.L., Corriveau E.J., Liem A.T., Willis K.L., Kadavy D.R., Rosenzweig C.N., Minot S.S. (2015). Bacterial and viral identification and differentiation by amplicon sequencing on the MinION nanopore sequencer. GigaScience.

[32] Walter M.C., Zwirglmaier K., Vette P., Holowachuk S.A., Stoecker K., Genzel G.H., Antwerpen M.H. (2017). MinION as part of a biomedical rapidly deployable laboratory. J. Biotechnol..

[33] Visser M., Maree H.J., Rees D.J.G., Burger J.T. (2014). High-throughput sequencing reveals small RNAs involved in ASGV infection. BMC Genom..

[34] Liang P., Navarro B., Zhang Z., Wang H., Lu M., Xiao H., Wu Q., Zhou X., Di Serio F., Li S. (2015). Identification and characterization of a novel geminivirus with a monopartite genome infecting apple trees. J. Gen. Virol..

[35] Chiumenti M., Morelli M., De Stradis A., Elbeaino T., Stavolone L., Minafra A. (2016). Unusual genomic features of a badnavirus infecting mulberry. J. Gen. Virol..

[36] Ruiz-García A.B., Martínez C., Santiago R., García M.T., de Prado N., Olmos A. (2016). First report of *Little cherry virus 1* (LChV-1) in sweet cherry in Spain. Plant. Dis..

[37] Ruiz-García A.B., Chamberland N., Martínez C., Massart S., Olmos A. (2017). First report of *Persimmon cryptic virus* in Spain. J. Plant Pathol..

[38] He Y., Cai L., Zhou L., Yang Z., Hong N., Wang G., Li S., Xu W. (2017). Deep sequencing reveals the first fabavirus infecting peach. Sci. Rep..

[39] Giampetruzzi A., Chiumenti M., Minafra A., Saldarelli P., Pantaleo V., Chiumenti M. (2018). Small RNA isolation from tissues of grapevine and woody plants. Viral Metagenomics; Methods in Molecular Biology.

[40] Koloniuk I., Sarkisova T., Petrzik K., Lenz O., Přibylová J., Fránová J., Špak J., Lotos L., Beta C., Katsiani A. (2018). Variability studies of two *Prunus*-infecting fabaviruses with the aid of high-throughput sequencing. Viruses.

[41] Baráth D., Jaksa-Czotter N., Molnár J., Varga T., Balássy J., Szabó L.K., Kirilla Z., Tusnády G.E., Preininger É., Várallyay É. (2018). Small RNA NGS revealed the presence of *Cherry virus A* and *Little cherry virus 1* on apricots in Hungary. Viruses.

[42] Czotter N., Molnár J., Pesti R., Demián E., Baráth D., Varga T., Várallyay É., Pantaleo V., Chiumenti M. (2018). Use of siRNAs for diagnosis of viruses associated to woody plants in nurseries and stock collections. Viral Metagenomics, Methods in Molecular Biology.

[43] Candresse T., Marais A., Faure C., Gentit P. (2013). Association of *Little cherry virus 1* with the Shirofugen stunt disease and characterization of the genome of a divergent LChV1 isolate. Phytopathology.

[44] Elbeaino T., Giampetruzzi A., De Stradis A., Digiaro M. (2014). Deep-sequencing analysis of an apricot tree with vein clearing symptoms reveals the presence of a novel betaflexivirus. Virus Res..

[45] Marais A., Faure C., Mustafayev E., Barone M., Alioto D., Candresse T. (2015). Characterization by deep sequencing of *Prunus virus T*, a novel Tepovirus infecting Prunus species. Phytopathology.

[46] Marais A., Faure C., Candresse T. (2016). New insights into Asian prunus viruses in the light of NGS-based full genome sequencing. PLoS ONE.

[47] Marais A., Faure C., Bergey B., Candresse T., Pantaleo V., Chiumenti M. (2018). Viral double-stranded RNAs (dsRNAs) from plants: Alternative nucleic acid substrates for high-throughput sequencing. Viral Metagenomics, Methods in Molecular Biology.

[48] Lenz O., Přibylová J., Fránová J., Koloniuk I., Špak J. (2017). Identification and characterization of a new member of the genus *Luteovirus* from cherry. Arch. Virol..

[49] Villamor D.E.V., Pillai S.S., Eastwell K.C. (2017). High throughput sequencing reveals a novel fabavirus infecting sweet cherry. Arch. Virol..

[50] Kesanakurti P., Belton M., Saeed H., Rast H., Boyes I., Rott M. (2017). Comparative analysis of cherry virus A genome sequences assembled from deep sequencing data. Arch. Virol..

[51] Dodds J.A., Morris M.T., Jordan R.L. (1984). Plant viral double-stranded RNA. Ann. Rev. Phytopathol..

[52] Loconsole G., Saldarelli P., Doddapaneni H., Savino V., Martelli G.P., Saponari M. (2012). Identification of a single-stranded DNA virus associated with citrus chlorotic dwarf disease, a new member in the family *Geminiviridae*. Virology.

[53] Coetzee B., Freeborough M.J., Maree H.J., Celton J.M., Rees D.J.G., Burger J.T. (2010). Deep sequencing analysis of viruses infecting grapevines: Virome of a vineyard. Virology.

[54] Jo Y., Choi H., Kyong C.J., Yoon J.Y., Choi S.K., Kyong C.W. (2015). In silico approach to reveal viral populations in grapevine cultivar Tannat using transcriptome data. Sci. Rep..

[55] Ruiz-García A.B., Olmos A. (2017). First Report of *Grapevine Pinot gris virus* in grapevine in Spain. Plant Dis..

[56] Ruiz-García A.B., Sabate J., Lloria O., Lavina A., Batlle A., Olmos A. (2017). First report of *Grapevine Syrah virus-1* in grapevine in Spain. Plant Dis..

[57] Jo Y., Choi H., Kim S.M., Kim S.L., Lee B.C., Cho W.K. (2016). Integrated analyses using RNA-Seq data reveal viral genomes, single nucleotide variations, the phylogenetic relationship, and recombination for *Apple Stem Grooving Virus*. BMC Genom..

[58] Roossinck M.J., Martin D.P., Roumagnac P. (2015). Plant virus metagenomics: Advances in virus discovery. Phytopathology.

[59] Wang Q., Jia P., Zhao Z. (2013). VirusFinder: Software for efficient and accurate detection of viruses and their integration sites in host genomes through next generation sequencing data. PLoS ONE.

[60] Jones S., Baizan-Edge A., MacFarlane S., Torrance L. (2017). viral diagnostics in plants using next generation sequencing: Computational analysis in practice. Front. Plant Sci..

[61] Loconsole G., Önelge N., Potere O., Giampetruzzi A., Bozan O., Satar S., De Stradis A., Savino V., Yokomi R.K., Saponari M. (2012). Identification and characterization of *Citrus yellow vein clearing virus*, a putative new member of the genus *Mandarivirus*. Phytopathology.

[62] Vives M.C., Velázquez K., Pina J.A., Moreno P., Guerri J., Navarro L. (2013). Identification of a new enamovirus associated with citrus vein enation disease by deep sequencing of small RNAs. Phytopathology.

[63] Roy A., Stone A., Otero-Colina G., Wei G., Choudhary N., Achor D., Shao J., Levy L., Nakhla M.K., Hollingsworth C.R. (2013). Genome assembly of *Citrus leprosis virus* nuclear type reveals a close association with *Orchid*. *Fleck Virus*. Genome Announc..

[64] Roy A., Hartung J.S., Schneider W.L., Shao J., Leon G., Melzer M.J., Beard J.J., Otero-Colina G., Bauchan G.R., Ochoa R. (2015). Role bending: Complex relationships between viruses, hosts, and vectors related to citrus leprosis, an emerging disease. Phytopathology.

[65] Roy A., Shao J., Hartung J.S., Schneider W., Brlansky R.H. (2013). A case study on discovery of novel *Citrus leprosis virus* cytoplasmic type 2 utilizing small RNA libraries by next generation sequencing and bioinformatic analyses. J. Data Min. Genom. Proteom..

[66] Roy A., Shao J., Schneider W.L., Hartung J.S., Brlansky R.H. (2014). Population of endogenous pararetrovirus genomes in Carrizo citrange. Genome Announc..

[67] Hartung J.S., Roy A., Fu S., Shao J.Y., Schneider W.L., Brlansky R.H. (2015). History and diversity of *Citrus leprosis virus* recorded in herbarium specimens. Phytopathology.

[68] Navarro B.L., Minutolo M., Stradis A.D., Palmisano F., Alioto D., Di Serio F. (2018). The first phlebo-like virus infecting plants: A case study on the adaptation of negative-stranded RNA viruses to new hosts. Mol. Plant Pathol..

[69] Matsumura E.E., Coletta-Filho H.D., Nouri S., Falk B.W., Nerva L., Oliveira T.S., Dorta S.O., Machado M.A. (2017). Deep sequencing analysis of RNAs from citrus plants grown in a citrus sudden death-affected area reveals diverse known and putative novel viruses. Viruses.

[70] Marais A., Faure C., Mustafayev E., Candresse T. (2015). Characterization of new isolates of *Apricot vein clearing-associated virus* and of a new *Prunus*-infecting virus: Evidence for recombination as a driving force in *Betaflexiviridae* evolution. PLoS ONE.

[71] Marais A., Faure C., Theil S., Candresse T. (2018). Molecular characterization of a novel species of Capillovirus from Japanese apricot (*Prunus mume*). Viruses.

[72] Wu L.P., Liu H.W., Bateman M., Liu Z., Li R. (2017). Molecular characterization of a novel luteovirus from peach identified by high-throughput sequencing. Arch. Virol..

[73] Bag S., Al Rwahnih M., Li A., Gonzalez A., Rowhani A., Uyemoto J.K., Sudarshana R. (2015). Detection of a new luteovirus in imported nectarine trees: A case study to propose adoption of metagenomics in post-entry quarantine. Phytopathology.

[74] Villamor D.E.V., Mekuria T.A., Pillai S.S., Eastwell K.C. (2016). High-throughput sequencing identifies novel viruses in Nectarine: Insights to the etiology of stem-pitting disease. Phytopathology.

[75] Villamor D.E., Susaimuthu J., Eastwell K.C. (2015). Genomic analyses of cherry rusty mottle group and cherry twisted leaf-associated viruses reveal a possible new genus within the family betaflexiviridae. Phytopathology.

[76] Igori D., Lim S., Baek D., Cho I.S., Moon J.S. (2017). Complete nucleotide sequence of a highly divergent cherry-associated luteovirus (ChALV) isolate from peach in South Korea. Arch. Virol..

[77] Igori D., Lim S., Baek D., Kim S.Y., Seo E., Cho I.S., Choi G.S., Lim H.S., Moon J.S. (2017). Complete nucleotide sequence and genome organization of Peach virus D, a putative new member of the genus *Marafivirus*. Arch. Virol..

[78] Jo Y., Lian S., Chu H., Cho J.K., Yoo S.H., Choi H., Yoon J.Y., Choi S.K., Lee B.C., Cho W.K. (2018). Peach RNA viromes in six different peach cultivars. Sci. Rep..

[79] Cho I.-S., Igori D., Lim S., Choi G.-S., Hammond J., Lim H.-S., Moon J.S. (2016). Deep sequencing analysis of apple infecting viruses in Korea. Plant Pathol. J..

[80] Morelli M., Giampetruzzi A., Laghezza L., Catalano L., Savino V.N., Saldarelli P. (2017). Identification and characterization of an isolate of apple green crinkle associated virus involved in a severe disease of quince (*Cydonia oblonga*, Mill.). Arch. Virol..

[81] Noda H., Yamagishi N., Yaegashi H., Xing F., Xie J., Li S., Zhou T., Ito T., Yoshikawa N. (2017). Apple necrotic mosaic virus, a novel ilarvirus from mosaic-diseased apple trees in Japan and China. J. Gen. Plant Pathol..

[82] Jakovljevic V., Otten P., Berwarth C., Jelkmann W. (2016). Analysis of the apple rubbery wood disease by next generation sequencing of total RNA. Eur. J. Plant Pathol..

[83] Rott M.E., Kesanakurti P., Berwarth C., Rast H., Boyes I., Phelan J., Jelkmann W. (2018). Discovery of negative-sense RNA viruses in trees infected with apple rubbery wood disease by next-generation sequencing. Plant Dis..

[84] Shen P., Tian X., Zhang S., Ren F., Li P., Yu Y., Li R., Zhou C.Y., Cao M. (2018). Molecular characterization of a novel luteovirus infecting apple by next-generation sequencing. Arch. Virol..

[85] Ito T., Suzaki K., Nakano M.J. (2013). Genetic characterization of novel putative rhabdovirus and dsRNA virus from Japanese persimmon. Gen. Virol..

[86] Morelli M., Chiumenti M., De Stradis A., La Notte P., Minafra A. (2015). Discovery and molecular characterization of a novel cryptovirus from Japanese persimmon through conventional cloning and high-throughput sequencing. Virus Genes.

[87] Ma Y., Navarro B., Zhang Z., Lu M., Zhou X., Chi S., Di Serio F., Li S.J. (2015). Identification and molecular characterization of a novel monopartite geminivirus associated with mulberry mosaic dwarf disease. J. Gen. Virol..

[88] Wang Y., Zhuang H., Yang Z., Wen L., Wang G., Hong N. (2018). Molecular characterization of an *Apple stem grooving virus* isolate from kiwifruit (*Actinidia chinensis*) in China. Can. J. Plant Pathol..

[89] Zheng Y., Navarro B., Wang G., Wang Y., Yang Z., Xu W., Zhu C., Wang L., Serio F.D., Hong N. (2017). Actinidia chlorotic ringspot-associated virus: A novel emaravirus infecting kiwifruit plants. Mol. Plant Pathol..

[90] Veerakone S., Liefting L.W., Tang J., Ward L.I. (2018). The complete nucleotide sequence and genome organisation of a novel member of the family *Betaflexiviridae* from *Actinidia Chinensis*. Arch. Virol..

[91] Barrero R.A., Napier K.R., Cunnington J., Liefting L., Keenan S., Frampton R.A., Szabo T., Bulman S., Hunter A., Ward L. (2017). An internet-based bioinformatics toolkit for plant biosecurity diagnosis and surveillance of viruses and viroids. BMC Bioinform..

[92] Zablocki O., Pietersen G. (2014). Characterization of a novel citrus tristeza virus genotype within three cross-protecting source GFMS12 sub-isolates in South Africa by means of Illumina sequencing. Arch. Virol..

[93] Read D., Pietersen G. (2017). Diversity of *Citrus tristeza virus* populations in commercial grapefruit orchards in Southern Africa, determined using Illumina MiSeq technology. Eur. J. Plant Pathol..

[94] Visser M., Cook G., Burger J.T., Maree H.J. (2017). In silico analysis of the grapefruit sRNAome, transcriptome and gene regulation in response to CTV-CDVd co-infection. Virol. J..

[95] Jooste T., Visser M., Cook G., Burger J.T., Maree H.J. (2017). in silico probe-based detection of citrus viruses in NGS data. Phytopathology.

[96] Licciardello G., Scuderi G., Ferraro R., Giampetruzzi A., Marcella R., Lombardo A., Raspagliesi D., Bar-Joseph M., Catara A. (2015). Deep sequencing and analysis of small RNAs in sweet orange grafted on sour orange infected with two *Citrus tristeza virus* isolates prevalent in Sicily. Arch. Virol..

[97] Nakazono-Nagaoka E., Fujikawa T., Iwanami T. (2017). Nucleotide sequences of Japanese isolates of citrus vein enation virus. Arch. Virol..

[98] Yu Y., Wu Q., Su H., Wang X., Cao M., Zhou C. (2017). Small RNA deep sequencing reveals full-length genome of *Citrus yellow vein clearing virus* in Chongqing, China. J. Integr. Agric..

[99] Kinoti W.M., Constable F.E., Nancarrow N., Plummer K.M., Rodoni B. (2017). Generic amplicon deep sequencing to determine *Ilarvirus* species diversity in Australian *Prunus*. Front. Microbiol..

[100] Marais A., Faure C., Couture C., Bergey B., Gentit P., Candresse T. (2014). Characterization by deep sequencing of divergent *Plum bark necrosis stem pitting-associated virus* (PBNSPaV) isolates and development of a broad-spectrum PBNSPaV detection assay. Phytopathology.

[101] Wang J., Zhai Y., Liu W., Zhu D., Pappu H.R., Liu Q. (2016). Complete genomic characterization of *Plum bark necrosis stem pitting–associated virus* infecting sweet cherry in China. Genome Announc..

[102] Lim S., Igori D., Yoo R.H., Zhao F., Cho I.S., Choi G.S., Lim H., Lee S.H., Moon J.S. (2015). Genomic detection and characterization of a Korean isolate of *Little cherry virus 1* sampled from a peach tree. Virus Genes.

[103] Wang J., Zhu D., Tan Y., Zong X., Wei H., Hammond R.W., Liu Q. (2016). Complete nucleotide sequence of *Little cherry virus 1* (LChV-1) infecting sweet cherry in China. Arch. Virol..

[104] Koinuma H., Nijo T., Iwabuchi N., Yoshida T., Keima T., Okano Y., Maejima K., Yamaji Y., Namba S. (2016). First complete genome sequence of *Cherry virus A*. Genome Announc..

[105] Wang J., Zhai Y., Liu W., Dhingra A., Pappu H.R., Liu Q. (2016). Structure and genome organization of *Cherry virus A* (*Capillovirus*, *Betaflexiviridae*) from China using small RNA sequencing. Genome Announc..

[106] Špak J., Přibylová J., Šafářová D., Lenz O., Koloniuk I., Navrátil M., Fránová J., Špaková V., Paprštein F. (2017). Cherry necrotic rusty mottle and Cherry green ring mottle viruses in Czech cherry germplasm. Plant Prot. Sci..

[107] Kinoti W.M., Constable F.E., Nancarrow N., Plummer K.M., Rodoni B. (2017). Analysis of intra-host genetic diversity of *Prunus necrotic ringspot virus* (PNRSV) using amplicon next generation sequencing. PLoS ONE.

[108] Kinoti W.M., Constable F.E., Nancarrow N., Plummer K.M., Rodoni B. (2018). The incidence and genetic diversity of *Apple mosaic virus* (ApMV) and *Prune dwarf virus* (PDV) in *Prunus* species in Australia. Viruses.

[109] Varveri C., Olmos A., Pina J.A., Marroquín C., Cambra M. (2015). Biological and molecular characterization of a distinct Citrus tristeza virus isolate originating from a lemon tree in Greece. Plant Pathology.

[110] Katsiani A., Maliogka V.I., Katis N., Svanella-Dumas L., Olmos A., Ruiz-García A.B., Marais A., Faure C., Theil S., Lotos L. (2018). High-throughput sequencing reveals further diversity of *Little Cherry Virus 1* with Implications for Diagnostics. Viruses.

[111] Al Rwahnih M., Daubert S., Golino D., Rowhani A. (2009). Deep sequencing analysis of RNAs from a grapevine showing Syrah decline symptoms reveals a multiple virus infection that includes a novel virus. Virology.

[112] Maliogka V.I., Olmos A., Pappi P.G., Lotos L., Efthimiou K., Grammatikaki G., Candresse T., Katis N.I., Avgelis A.D. (2015). A novel grapevine badnavirus is associated with the Roditis leaf discoloration disease. Virus Res..

[113] García J.A., Glasa M., Cambra M., Candresse T. (2014). *Plum pox virus* and sharka: A model potyvirus and a major disease. Mol. Plant Pathol..

[114] Katsiani A.T., Maliogka V.I., Amoutzias G.D., Efthimiou K.E., Katis N.I. (2015). Insights into the genetic diversity and evolution of *Little Cherry Virus 1*. Plant Pathol..

[115] Aiewsakun P., Katzourakis A. (2015). Endogenous viruses: Connecting recent and ancient viral evolution. Virology.

[116] Martin R., Zhou J., Tzanetakis I. (2011). Blueberry latent virus: An amalgam of the *Partitiviridae* and *Totiviridae*. Virus Res..

[117] Osaki H., Sasaki A., Nakazono-Nagaoka E., Ota N., Nakaune R. (2017). Genome segments encoding capsid protein-like variants of *Pyrus Pyrifolia Cryptic Virus*. Virus Res..

[118] Massart S., Candresse T., Gil J., Lacomme C., Predajna L., Ravnikar M., Reynard J.-S., Rumbou A., Saldarelli P., Škoric D. (2017). A Framework for the evaluation of biosecurity, commercial, regulatory, and scientific impacts of plant viruses and viroids identified by NGS technologies. Front. Microbiol..

[119] Candresse T., Filloux D., Muhire B., Julian C., Galzi S., Fort G., Bernardo P., Daugrois J.-H., Fernandez E., Martin D.P. (2014). Appearances can be deceptive: Revealing a hidden viral infection with deep sequencing in a plant quarantine context. PLoS ONE.

[120] Melcher U., Muthukumar V., Wiley G.B., Min B.E., Palmer M.W., Verchot-Lubicz J., Ali A., Nelson R.S., Roe B.A., Thap V. (2008). Evidence for novel viruses by analysis of nucleic acids in virus-like particle fractions from *Ambrosia Psilostachya*. J. Virol. Methods.

[121] Basso M.F., da Silva J.C., Fajardo T.V., Fontes E.P., Zerbini F.M. (2015). A novel, highly divergent ssDNA virus identified in Brazil infecting apple, pear and grapevine. Virus Res..

[122] Fontenele R.S., Abreu R.A., Lamas N.S., Alves-Freitas D.M.T., Vidal A.H., Poppiel R.R., Melo F.L., Lacorte C., Martin D.P., Campos M.A. (2018). *Passion fruit chlorotic mottle virus*: Molecular characterization of a new divergent geminivirus in Brazil. Viruses.

